# Microglia sequelae: brain signature of innate immunity in schizophrenia

**DOI:** 10.1038/s41398-022-02197-1

**Published:** 2022-11-28

**Authors:** A. Catarina Rodrigues-Neves, António. F. Ambrósio, Catarina A. Gomes

**Affiliations:** 1 Univ Coimbra, Coimbra Institute for Clinical and Biomedical Research (iCBR), Faculty of Medicine, Coimbra, Portugal; 2 Univ Coimbra, Center for Innovative Biomedicine and Biotechnology (CIBB), Coimbra, Portugal; 3 Clinical Academic Center of Coimbra (CACC), Coimbra, Portugal; 4 Univ Coimbra, Faculty of Pharmacy, Coimbra, Portugal

**Keywords:** Molecular neuroscience, Schizophrenia

## Abstract

Schizophrenia is a psychiatric disorder with significant impact on individuals and society. The current pharmacologic treatment, which principally alleviates psychosis, is focused on neurotransmitters modulation, relying on drugs with severe side effects and ineffectiveness in a significant percentage of cases. Therefore, and due to difficulties inherent to diagnosis and treatment, it is vital to reassess alternative cellular and molecular drug targets. Distinct risk factors – genetic, developmental, epigenetic, and environmental – have been associated with disease onset and progression, giving rise to the proposal of different pathophysiological mechanisms and putative pharmacological targets. Immunity is involved and, particularly microglia – innate immune cells of the central nervous system, critically involved in brain development – have captured attention as cellular players. Microglia undergo marked morphologic and functional alterations in the human disease, as well as in animal models of schizophrenia, as reported in several original papers. We cluster the main findings of clinical studies by groups of patients: (1) at ultra-high risk of psychosis, (2) with a first episode of psychosis or recent-onset schizophrenia, and (3) with chronic schizophrenia; in translational studies, we highlight the time window of appearance of particular microglia alterations in the most well studied animal model in the field (maternal immune activation). The organization of clinical and translational findings based on schizophrenia-associated microglia changes in different phases of the disease course may help defining a temporal pattern of microglia changes and may drive the design of novel therapeutic strategies.

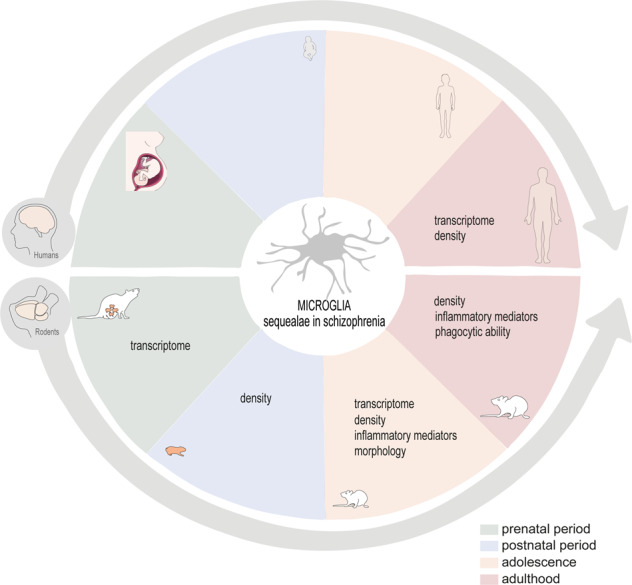

## Introduction

**Fig. 1:**
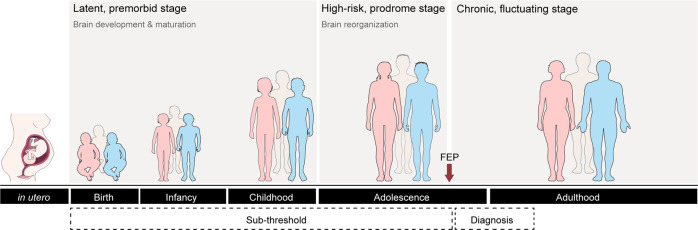
Schizophrenia progression involves three stages. (1) latent, pre-morbid (from the prenatal period until childhood) implicates biological processes intrinsic to brain development and maturation; (2) high-risk, prodromal (usually initiates in adolescence) - impacts on brain reorganization (e.g. sexual maturation); and (3) chronic, fluctuating (adulthood).

Schizophrenia is a chronic multifactorial mental disorder, presenting variable clinical manifestations. Patients manifest different symptoms, classified as positive (e.g. delusions, hallucinations), negative (e.g. social withdrawal, self-neglect, avolition, and loss of motivation) and/or cognitive (e.g. working memory and attention deficits) [1]. The first symptoms usually manifest during late adolescence and early adulthood and are, in the majority of cases, depressive and negative symptoms (in a minority of cases, psychotic symptoms are the first to appear) [2]. Due to the scarce symptomatology until the occurrence of the first episode of psychosis, if we consider the chronopathology of schizophrenia, the diagnosis is late and patients already present substantial brain changes, in terms of structure, neurochemistry, and connectivity [3]. Based on schizophrenia onset and progression, three stages are established: (1) latent, pre-morbid – implicates biological processes intrinsic to brain development and maturation and extends from the prenatal period to childhood; (2) high-risk, prodromal – usually initiates in adolescence, hampering brain reorganization (e.g. sexual maturation) and (3) chronic, fluctuating – occurs at adulthood (for more details see ref. [3]; Fig. 1).

**Fig. 2:**
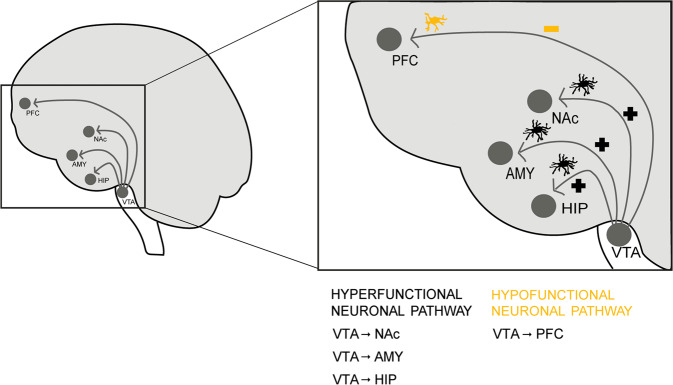
Dopamine hypothesis underlying schizophrenia pathophysiology (hypo- and hyperfunctional dopaminergic pathways). Schematic representation of hypofunctional (decreased dopamine transmission to the prefrontal cortex (PFC)) and hyperfunctional (increased dopamine transmission to nucleus accumbens (NAc), amygdala (AMY) and hippocampus (HIP).

In addition to the diversity in clinical presentation, the identified risk factors (from development and genetic to environmental and epigenetic) also reflect the involvement of several biological systems in the genesis and progression of the disease [4, 5]. In consequence, several theories (focused on environmental and genetic factors, neurochemical and neuroanatomical abnormalities [6–8]) have emerged in an attempt to clarify the biological basis of schizophrenia. Objectively, if we consider the drugs currently used in the clinical practice (different generations of modulators of the subtype D2 of dopamine receptors, modified over the years in order to attenuate severe side effects associated with D2 receptor blockade [9]), dopamine hypothesis is the most well accepted theory. It postulates an (1) excess of dopamine transmission in subcortical limbic areas, such as the nucleus accumbens (NAc), amygdala (AMY), and hippocampus (HIP) (mesolimbic pathway) – responsible for the positive symptoms [10, 11] - and a (2) dopamine deficiency in the prefrontal cortex (PFC; mesocortical pathway) – responsible for the negative and cognitive symptoms [12] (Fig. 2). Despite the unquestionable beneficial effects, antipsychotics still present important limitations, including behavioral, neurological (namely extrapyramidal effects), hematological, cardiovascular, metabolic, and endocrine (weight gain, hyperlipidemia, and diabetes mellitus) adverse effects [7, 13–16]. The identification of alternative cellular and molecular drug targets would fill the gap that still remains in schizophrenia treatment.

Over the past years, immunity has been implicated in the genesis, progression and clinical manifestations of psychiatric disorders, including schizophrenia (for review [17–20]). Although out of the scope of this review, we cannot afford to mention the immunomodulatory properties of antipsychotic drugs [21, 22], namely the ability to normalize altered levels of inflammatory mediators, reported as a mechanism underlying the remission of psychotic symptoms [23].

Innate immunity involves different cells able to mount an inflammatory response to insults, including microglia, which colonize the central nervous system during early neurodevelopment. Throughout life and from the very beginning of brain development, these innate immune cells perform an essential function – synapse pruning, which involves the phagocytosis of synapses [24–26]. In the past, it has been proposed that an aberrant synaptic pruning occurs in schizophrenia [4, 27–29], raising the idea that microglia could be involved in its genesis and progression. Notably, the effect of antipsychotics on microglia (for a review see ref. [30]) is not well established, a nuclear question to clarify if drug efficacy is related with the modulation of disease-associated microglia sequelae. The aim of this review is not to summarize the inflammatory-immune hallmarks of schizophrenia, but rather focused on disease-associated changes of this specific cellular candidate – microglia. These cells are in tight communication with the first newborn neurons, being sensitive and responsive to neurochemical signs (reviewed in refs. [31, 32]), exerting a crucial modulatory role in the formation/maturation of neurons and neuronal circuits in critical periods of brain development, and later, contributing to brain homeostasis. We here gather clinical and translational findings related with microglial changes (sequelae) in schizophrenia.

## Microglia in brain development and homeostasis: kickoff to schizophrenia

Microglia are immune cells derived from embryonic yolk-sac myeloid progenitors. After the colonization of the brain (early in the embryonic period, before the completion of blood-brain barriers) [33], microglia migrate towards different brain regions, proliferate and mature at different paces from species to species. After the initial colonization, self-renewal by proliferation is accepted as the only mechanism of repopulation throughout life [34].

These heterogeneous cells are categorized in subpopulations, according to the expression of particular gene clusters, and present distinct transcriptomic profiles with brain region specificity, that change over time to functionally respond to particular demands of different developmental phases [34–36]. Microglia also present a panoply of morphologic phenotypes, ranging from amoeboid to varied degrees of cellular processes ramification [37]. In response to different stimuli, a notable ability to change from amoeboid to ramified phenotypes (and vice-versa) is granted by cellular processes well adapted to the surveillance of brain parenchyma, through dynamic extension and retraction mechanisms [38, 39]. Importantly, microglia do not transit only between a “healthy” ramified and a “pathological” amoeboid phenotype, as initially believed. Instead, microglia undergo morphological adaptions, depending on their brain location and stimuli, a topic that our group has been studying in the context of mental diseases [40–42].

Due to their phagocytic ability and capacity to produce and release inflammatory mediators and growth factors, microglia actively contribute to the proper establishment and maturation of neural circuits. Microglia can stimulate neurogenesis by supporting survival, proliferation, and maturation of neuronal progenitor cells (NPCs) and neurons [43]) or, instead, eliminate neurons, NPCs or apoptotic cells by phagocytosis (reviewed in refs. [32, 44]).

The settlement between elimination or strengthening depends on microglia-neuron communication, that occurs through the release of soluble factors [45] or physical contact [31, 46]. Distinct signaling pathways, involving fractalkine/fractalkine receptor (CX3CL1/CX3CR1), OX-2 membrane glycoprotein/OX-2 membrane glycoprotein receptor (CD200/CD200R), Toll-like receptors (TLRs), cytokines, complement pathways (complement component 1q/complement component 1q receptor – C1q/C1qR, complement component 3/complement component 3 receptor – C3/C3R), extracellular vesicles, among others [47–49], mediate neuron-microglia interactions.

Microglia is able to eliminate or strengthen cells and subcellular domains, namely synapses [24], a function of high relevance in the onset of schizophrenia, proposed to be coincident with an aberrant process of synapse pruning [50, 51], as firstly reported by Feinberg [52]. In schizophrenia, the complement system is a key intervenient in synapse elimination by microglia, in particular the components C4 and C3. The former, which is increased in the hippocampus of patients (in neurons and synapses) [53], activates C3, that binds to a receptor exclusively expressed by myeloid cells, including microglia [26, 54, 55]. Other authors demonstrated that C4 variants associated with schizophrenia, increase C3 deposition in neurons [51], synaptic engulfment by microglia [51, 56] and reduce the connectivity in the PFC of prepubertal rats [56].

This group of findings clearly points to the involvement of the complement pathway, further supporting the proposed theory of excessive synaptic pruning in the genesis of schizophrenia [50]. If this represents one of the triggering events of the disease, the pharmacologic modulation of the complement system emerges as a potential therapeutic approach. However, as previously mentioned, diagnosis is usually subsequent to the first episode of psychosis, when substantial changes already occurred in the brain, including cellular plastic events of microglia, eventually contributing to disease progression. To clearly define microglia as a cellular target in the disease, one must know the nature and the time of appearance of specific microglial changes with disease progression. In the following sections, we reunite data from clinical and non-clinical studies focused on microglia sequelae.

## Microglia sequelae: clinical findings in SCZ patients

Several epidemiological studies have shown that changes in the immune system are linked to psychosis and, therefore, to schizophrenia pathophysiology [57–60]. Most studies describe alterations in cellular and/or molecular mediators assessed in patient blood, rather than immune changes in the brain. The detection of peripheral immune changes is a more feasible and low-cost approach, that may help finding novel biomarkers of disease, improving diagnosis and progression monitoring. For instance, a correlation was established between increased levels of interleukine-6 in serum (measured at 9 years old) with a twofold increase risk of developing psychotic symptoms at 18 years [61]. Although aware of the importance of peripheral immune changes that may have the contribution of factors produced and released by microglia (properly reviewed elsewhere, e.g. [17–20]), the main goal of the present article is to review changes exclusively related with this particular type of immune cells of the central nervous system.

Here, we organized the clinical studies in two subsections, neuroimaging techniques, that allow in vivo monitorization and longitudinal analyses, and post-mortem assessments, a complementary approach that helps finding molecular and cellular pathophysiological traits of the disease.

### Neuroimaging approaches as a readout for neuroinflammation mediated by microglia

In the last years, new techniques and methodologies granted significative advances in neuroimaging. Even though, there is a lack of reliable markers to properly evaluate microglia. The available methods mainly target a mitochondrial protein (18-kDa translocator protein, TSPO) highly expressed in activated microglia, that is, involved in an inflammatory response to pathological conditions, such as psychiatric diseases [62]. Thus, the measurement of TSPO levels through the use of radiotracers has been commonly performed to investigate microglia activation in SCZ patients [63], although we would like to emphasize that TSPO is not exclusively expressed in microglial cells [64] and its expression does not correlate with the expression of canonical activation markers [65]. Anyway, we consider of importance to reunite and carefully analyze clinical findings based on TSPO evaluation to finally take objective conclusions about the validity of considering TSPO as a readout of microglia-mediated neuroinflammation (see Table 1).

**Table 1:** Microglia sequelae in schizophrenia patients (evidences from neuroimaging studies based in TSPO measurement).

		Radiotracer	Brain region	TSPO finding	Refs.
Patients at ultra-high risk (UHR) of psychosis	–	^11^C-PK11195	Dorsal frontal, orbital frontal, anterior cingulate, medial temporal, thalamus, and insula	No changes	[67]
	–	^11^C-PBR28	Total gray matter, frontal, and temporal lobe	Increase	[75]
Patients with FEP or recent-onset schizophrenia (includes acute relapse patientsa)	FEP	[^18^F]FEPPA	Dorsolateral and medial prefrontal cortex, temporal cortex, hippocampus, total gray matter, and whole brain	No changes	[73]
	FEP	^11^C-PBR28	Gray matter, white matter, frontal and temporal cortex, and hippocampus	Decrease	[74]
	FEP	[^18^F]PBR111	Cortical lobes and cingulated cortex, cerebellum, brain stem, thalamus, basal ganglia, amygdala, and hippocampus	Increase^a^	[76]
	Recent-onset	^11^C-PK11195 ^11^C-PK11195 ^11^C-PK11195 [^11^C]DPA-713	Dorsal frontal, orbital frontal, anterior cingulate, medial temporal cortices, thalamus, and insula; anterior cingulate, prefrontal, orbitofrontal, parietal and temporal cortices, caudate, putamen, thalamus, amygdala, hippocampus, and brainstem; frontal, temporal and parietal cortices, thalamus, and striatum; Cingulate, parietal, frontal, temporal and occipital cortices, hippocampus, and amygdala	No changes	[67, 69, 70, 77]
	Recent-onset	^11^C-PK11195	Total gray matter (all brain)	Increase	[71]
Patients with well-established schizophrenia	–	^11^C-PK11195 ^11^C-PK11195 [^18^F]FEPPA	Dorsal frontal, orbital frontal, anterior cingulate, medial temporal, thalamus, and insula; anterior cingulate, prefrontal, orbitofrontal, parietal and temporal cortices, caudate, putamen, thalamus, amygdala, hippocampus, and brainstem; Medial and dorsolateral prefrontal cortices, temporal cortex, hippocampus, and striatum	No changes	[67, 69, 78]
	–	^11^C-PK11195 ^11^C-PK11195 ^11^C-PBR28	Dorsolateral and ventrolateral prefrontal cortices, orbitofrontal, anterior cingulate, and parietal cortices; Frontal, temporal, parietal and occipital cortices, basal ganglia, hippocampus, and cerebellum; Total gray matter, frontal, and temporal lobe	Increase	[68, 72, 75]

^a^Includes acute relapse patients.

To better understand the potential conflicting results between studies, it is important to consider some bias related to TSPO radiotracers, starting from the binding affinity of the radioligands, that ranges from low (for first generation radiotracers) to high (for second-generation radiotracers), which potentially influences the analysis. Besides, the binding of some radiotracers is conditioned by a common genetic polymorphism in exon 4 of the TSPO gene (rs6971), resulting in distinct TSPO binding profiles (for more details see ref. [66]). The methods applied to calculate total/regional TSPO binding also vary among studies: simplified reference tissue model (method mostly used for 11C-PK11195 radiotracer) [67–70]; two-tissue component model (2TCM) with metabolite-corrected arterial plasma curve as input function (11C-PK11195 [71, 72] or second-generation radiotracer [64, 71–78]) and/or two-tissue compartmental model accounting for endothelial vascular TSPO binding (2TCM-1K) [74–76, 79]. Finally, schizophrenia itself accounts for important confounding factors, such as cohorts heterogeneity, medication, stage of disease progression, to mention a few (see review [80]).

For the sake of clarity, we divided neuroimaging studies in three groups of patients in different stages of the disease: (1) ultra-high risk of psychosis, (2) first episode of psychosis (FEP) or recent-onset schizophrenia and (3) chronic schizophrenia.

#### Patients at ultra-high risk of psychosis

In the literature, we only found two studies exploring TSPO binding in patients at ultra-high risk of psychosis. Curiously, normal values of TSPO tracing were described when a low binding affinity radiotracer (^11^C-PK11195) was used (the low affinity may hypothetically limit the detection of subtle changes) [67]. When a second-generation radiotracer (^11^C-PBR28) was used, the authors established a correlation between increased TSPO tracing and the severity of symptoms [75]. It is important to emphasize that, in addition to differences in the affinity of radiotracers and methodologic approaches, the conclusions were supported by observations in different brain regions.

#### Patients with FEP or recent-onset schizophrenia

As described in patients at risk of psychosis, in this group of patients with FEP, the results are conflicting, with three main studies describing an increase [76], a decrease [74] or the absence of changes [73] in TSPO tracing. Again, besides differences in radiotracer affinities, the heterogeneity of the individuals involved in the studies may influence the results, as occurs with the study reporting an increase in TSPO [76], that includes patients with an acute relapse, that likely present exacerbated microglia-mediated neuroinflammation. The complexity of the analysis increases if we segregate patients under medication and not treated, since pharmacotherapy is an important confounding effect, mainly considering the immune modulatory effect of antipsychotics, as previously mentioned. Normal TSPO was observed in a cohort under antipsychotic medication [73], whereas decreased TSPO levels were found in non-treated FEP patients [74] or recent-onset schizophrenia [69].

In patients with recent-onset schizophrenia under antipsychotic treatment, most studies indicate the absence of neuroinflammation, as assessed by TSPO evaluation [67, 69, 70, 77]. Even though, we were able to find two studies reporting abnormal TSPO levels [64, 71].

#### Patients with chronic schizophrenia

Studies including patients with chronic schizophrenia, that are usually under antipsychotic treatment, report increases [68, 72, 75] or normal levels of TSPO [67, 69, 78]. In this case, one cannot justify the absence of TSPO changes with the use of low affinity radiotracers, since the use of the low affinity ^11^C-PK11195 radiotracer did not allow the detection of changes in two studies [67, 69], but detected increased TSPO expression in another study/cohort [72].

In summary, the available published studies applying TSPO tracing, are not sufficient to correlate disease progression and/or severity of symptoms with microglia-mediated inflammation. One cannot exclude the existence of a correlation, simply because the studies were not designed taking into account the influence of strong bias, from the stage of the disease, the severity of symptoms or the influence of medication (a problem even more important if we consider that different classes of drugs with potential different immune modulatory effects exist). Efforts have been made to circumvent this heterogeneity, increasing the level of patient stratification; however, stratification based on highly selective criteria implies the inclusion of higher number of individuals, which constitutes a serious limitation in clinical practice.

### Schizophrenia-associated microglia alterations revealed by post-mortem studies

Post-mortem analysis is an important tool to identify and characterize changes in microglia that are not possible to detect in live patients, although one must be aware of important confounding factors, namely the cause of death, co-morbidities, medication, lack of knowledge of disease stage, and/or symptoms predominance, to mention a few. Some studies do not mention the stage or the predominant symptomatology and only refer the involvement of patients with a diagnosis [65, 81–83] or chronic schizophrenia [84–88]. The lack of information about disease staging led us to organize post-mortem data by brain region (as a summary, please see Table 2).

**Table 2:** Microglia sequelae in schizophrenia patients (post-mortem evidences).

Brain region		Finding	Changes	Refs.
Prefrontal cortex	–	Transcripts (TSPO, CX3CR1, ITGAM, CD163, HLA-DRA, IL-1β levels)^b^	No changes	[65]
		Microglia density	No changes	[81, 82]
			Increase	[84–86, 92, 94]
		Morphologic profile	Increase soma size and decrease arborization	[86]
		Phagocytic function	No changes	[87]
Cingulate cortex	–	Total number of microglia cells (lateralization effect)	High density of microglia in right hemisphere than the left	[83]
Temporal cortex	–	Transcripts (CX3CR1, TMEM 119, P2Y12, CSFR1, among others)	Decrease	[82]
		Microglia density	Increase	[82, 84, 86]
Subcortical regions	Substantia nigra	Transcripts (AIF-1, CD68, and TSPO)|only in high immune group^a^	Increase^a^	[88]
	Corpus callosum	Transcripts (TSPO)^b^	No changes	[65]
	Hippocampus	Microglia density	Increase	[96]

^a^This increase was only detected in a high immune group

^b^All studies were performed in tissue with the exception of TSPO evaluation in prefrontal cortex and corpus callosum

Glia plasticity, in general, and microglia plasticity, in particular, have been a matter of debate in the fields of basic neurosciences and psychiatry/neurology. The concept refers to the ability of microglia to undergo adaptative changes at different levels, namely in cell density, morphology and/or function, in response to physiological or pathological stimuli. For instance, alterations in the number of microglial cells may reflect a proliferative status, that typically occurs in non-physiological conditions (with the exception of normal brain development, characterized in certain phases by intense microglia proliferation). The morphological remodeling of microglia is also a biologic finding of several diseases and, interestingly, pre-clinic studies point towards a correlation between symptoms amelioration and the recovery of the healthy, normal morphology of microglia by pharmacologic interventions [40, 89]. One of the advantages of post-mortem analyses is precisely the possibility of performing a characterization of the so-called disease-associated microglia (DAM), as described in the context of neurodegenerative diseases [90]. Typically, the presence of amoeboid microglia was taken for decades as an index of activation, usually implicated in the response to an injury or brain disease. So, several studies in the literature report microglia changes in schizophrenia patients, based on the dual analysis of two phenotypes, ramified (resting) or amoeboid (activated) cells. This approach has been gradually replaced by more sophisticated morphometric methodologies, that include the determination of morphologic parameters considered relevant for microglia function, such as the degree of ramification or the brain area occupied per cell (hypothetically related with the efficacy of immune surveillance). We have also contributed to the finding that microglia present distinct regional morphologic phenotypes in the brain and that the morphologic remodeling in pathologic conditions is also different according to the brain region under analysis [40–42, 89, 91].

Besides the characterization of microglia morphology, post-mortem studies allow the quantitative analysis of parameters that may reflect the functional state of microglia, namely the local levels of inflammatory mediators (although other cells in the brain are able to produce and release these substances), enzymes involved in the inflammatory response operated by microglia, among others, that will be presented throughout this and the next sections. The data obtained deserve careful interpretation, as the readouts of cellular physiology are, as expected, seriously affected by the process of cell death and the underlying lesion.

Considering the regional segregation of microglia, we will then review the main findings per brain region, in particular because schizophrenia-associated microglia phenotypes were recently described in schizophrenia patients (increase of microglia soma size and decrease of arborization) [86].

#### Prefrontal region

Starting with the analysis of data collected in the frontal cortex, several studies report an increase in the density of microglia [84–86, 92, 93]. We found one study where, in general, no changes were detected, but reporting an increase in the number of activated microglia in three patients (without convincing justification/discussion) [81]. One of the referred studies also demonstrates the increase of cytokine levels [93] and other the integrity of the phagocytic ability of microglia [87]. In opposition, a recent meta-analysis [82] stands for the absence of changes in microglia density in the frontal cortex from schizophrenia post-mortem tissue. Of note, this meta-analysis included all studies with patients with a diagnosis, without considering the disease stage, symptoms predominance and other bias, that must be taken in consideration, as suggested by a study claiming for a correlation between the increased number of amoeboid microglia and the predominance of positive symptoms [92]. Other factor that may feed the controversy is the effect of lateralization (differences between hemispheres); it was recently observed, although specifically in the cingulated cortex, a higher density of microglia in the right hemisphere, as compared with the left one [83]. Finally, some authors evaluate microglia transcripts in the frontal cortex (particularly in the middle frontal gyrus), but no changes were detected, namely in TSPO labeling or in genes associated with microglia in physiologic (Allograft inflammatory factor 1 - AIF1, Integrin alpha M - ITGAM) or activated state (Cluster of Differentiation 163 - CD163, HLA-DRA, interleukin (IL)-1β) [65]. Nevertheless, a down-regulation of CX3CR1 ligand (CX3CL1) [94], mainly released by neurons, and its receptor (CX3CR1), present in microglia [95], was found in this brain region, suggesting a compromise of neuron-microglia communication.

#### Temporal region

In the temporal region, the evidences gathered so far are consistent, converging for an increase of microglia density [82, 84, 86] accompanied by a decrease in several transcripts (e.g. CX3CR1, Transmembrane Protein 119 - TMEM 119, Purinergic Receptor P2Y12 - P2RY12, HLA class II histocompatibility antigen, DR alpha chain - HLA-DRA, Colony stimulating factor 1 receptor - CSFR1) [82].

#### Subcortical regions

In subcortical regions, we found two reports about microglia transcripts in schizophrenia patients, one claiming for no changes in the corpus callosum [65] and the other showing an increase of several transcripts in the substantia nigra [88]. In the hippocampus, we found a very interesting finding on the relation between symptoms and microglia changes: the authors observe higher microglia numbers in patients with predominant psychotic/positive symptoms, as compared with patients with predominant negative symptoms [96]. This very relevant observation is clearly related with other finding by Purves-Tyson in 2019, that failed to find transcriptomic changes in schizophrenia patients until their stratification, in the case, by splitting the group in high and low immune biotypes [88]. The increased levels of AIF-1, CD68, and TSPO transcripts was only detectable in the subgroup of high immune biotype [88], whereas increased numbers of T and B lymphocytes were detected in schizophrenia with a different clinical presentation, categorized as paranoid [96], reinforcing the contribution of the peripheral immune system in particular contexts of the disease [17, 18]. In line with this concept, it was reported the activation of microglial cells in culture by exposure to serum samples from recent-onset schizophrenia patients [97], proving the responsiveness of microglia to peripheral signals and strengthening the interest of these cells as targets in the context of schizophrenia pharmacologic treatment.

## Microglia sequelae: findings from SCZ animal models

Animal models, although unable to replicate human diseases, are valuable tools in the study of neurobiological basis, mainly in the case of brain, that is of particularly difficult access. Despite the obvious limitations of animal models, such as the absence of symptoms of human schizophrenia (e.g., hallucinations, delusions), the fact is that several neurochemical changes, behavioral alterations, and biomarkers accomplish validity criteria to address pathophysiological mechanisms and to test novel therapeutic tools [98].

**Fig. 3:**
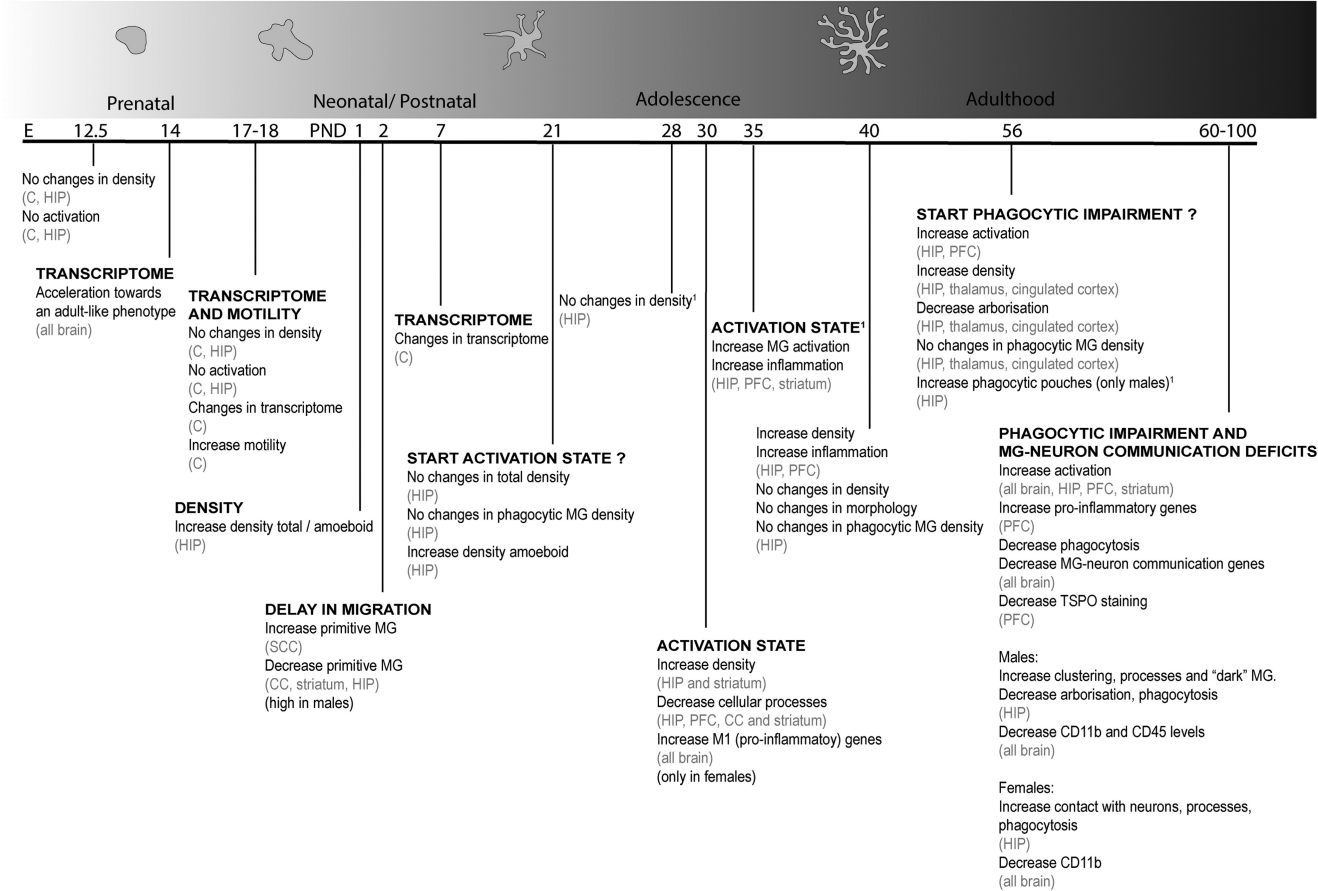
Microglia sequelae in schizophrenia animal models. The alterations are organized in four periods of development: prenatal, neonatal, adolescence and adulthood. Titles in capital letters evidence the main alteration(s) observed at a particular period of life. ED embryonic day, PND postnatal day, MG microglia, C cortex, HIP hippocampus, SCC supraventricular cortex, CC corpus callosum, PFC prefrontal cortex.?imprecise time window of appearance of microglia alterations. ^1^evidences from other SCZ animal models.

Although the etiology of schizophrenia is not fully understood, it is known that gestation is a period particularly sensitive, namely to genetic and environmental risk factors. In the literature, studies on microglia alterations in schizophrenia are mainly described in a model of maternal immune activation, the reason why this model is the most mentioned along this section. This animal model consists in the administration of an agonist of the Toll-like receptor 3, poly I:C (polyinosinic:polycytidylic acid) to pregnant rodents. Several protocols are found in the literature, namely concerning the gestational time window of administration (early gestation: 8–9.5 days; mild gestation: 12.5–13.5; mild-late gestation: 14.5–15 days; late gestation: 16.5–18.5 days). These differences in the protocol have a tremendous impact on brain development and require cautious interpretation of results. Adult descendants develop neuroanatomical, neurochemical and behavioral changes, some replicating disease traits found in schizophrenia patients, such as dopamine hyperactivity, ventricles enlargement, social and cognitive deficits and repetitive behaviors, that are clearly dependent of the gestational period of administration (for more details see [99]). In this article, we organize data related with microglia changes, mainly in poly I:C model (poly I:C - Table 3; others - Table 4), covering different periods of life (prenatal period, postnatal period, adolescence and adulthood; the main findings are summarized in Fig. 3). This chronological organization of microglia alterations (even aware of the importance of the time of exposure to poly I:C) may help identifying precise period(s) of microglia changes and, thus, of therapeutic modulation, hopefully as precociously as possible in the trajectory of the disease.

**Table 3:** Microglia sequelae: evidences from neurodevelopment until adulthood in poly I:C schizophrenia animal model.

Poly I:C animal model	Offspring age				

	Prenatal				
	ED 12.5	ED 14	ED 17	ED 17.5	ED 18
**CX3CR1-GFP mice** single (GD 11.5) or double (GD 11.5 and 15.5)	No changes in density nor in activation phenotype (cortex and hip) [105]	–	–	No changes in density nor in activation phenotype (cortex and hip) [105]	–
**C57BL/6J mice** single (GD 12.5 or 14.5)	–	Acceleration of microglia transcriptional profile towards to an adult-like phenotype (all brain) [34]	–	–	–
**C57BL6/J mice** single (GD 9.5)	–	–	Alterations in microglia transcriptome (genes involved in cell protrusion and neurogenesis) (cortex) [103]	–	–
**CX3CR1-GFP mice** single dose (GD12 or GD15)	–	–	–	–	Increased microglia motility No changes in density and morphology (cortex) [104]

Poly I:C animal model	Offspring age		

	Neonatal		
	PND 1	PND 2	PND 7
**Spiny mice** single (GD 20)	↑ Number of microglia ↓ of cellular processes (hip) [106]	–	–
**Sprague Dawley** single (GD 15)	–	↑ Primitive (amoeboid shape typical from early neurodevelopmental phases) microglia (SCC) (more in ♂ than ♀) ↓, Primitive microglia (CC, striatum, hip somatosensorial cortex) [107]	–
**C57BL6/J mice** single (GD 9.5)	–	–	Alterations in microglia transcriptome (cortex) [103]

Poly I:C animal model	Offspring age		

	Adolescence		
	PND 21	PND 30	PND 40
**C57BL6/N mice** single (GD 9)	Similar number of microglia (Iba-1^+^) and phagocytic microglia (CD68^+^) (hip) [110]	–	–
**Wistar rats** single (GD 15)	↑ Number of microglia amoeboid (hip) (only in ♂) [111]	–	–
**Balb/c mice** single (GD 9)	–	↑ Number of microglia (hip and striatum) ↓, Number of branches (activated microglia) (hip) [113]	–
**Balb/c mice** single (GD 9.5)	–	↑ Microglia activation (enlarged cell bodies and processes retraction) (hip, PFC, CC and striatum) [114]	–
**Balb/c mice** single (GD 9)	–	↑ Microglia activation (genes associated with M1 phenotype) (only in ♀) (all brain) [115]	–
**Sprague Dawley** single (GD 9)	–	–	↑ Number of microglia ↑ IL-1 p, IL-6 (PFC and hip) [117]
**C57BL6/N mice** single (GD 9)	–	–	Similar number of microglia, morphology and phagocytic microglia (CD68^+^ cells) p) [110]

Poly I:C animal model	Offspring age

	Adulthood
	PND 56-100
**Sprague Dawley** single (GD 9)	↑ Microglia activation (PFC and hip) [119]
**Sprague Dawley** single (GD 15)	↑ Microglia activation; ↑ microglia density; similar phagocytic microglia (thalamus, cingulated cortex and hip) [120]
**C57BL/6 mice** single (GD 12)	Microglia hyper-ramification (hip) Without changes in soma and primary branches [118]
**C57BL/6J mice** single (GD 9.5)	↑ Pro-inflammatory genes (PFC) [117, 125]
**C57BL/6 mice** single (GD 15)	↑ Microglia activation ↓ Microglia phagocytosis ↓ Genes related with microglia-neuron communication (all brain) [123]
**C57BL/6 mice** single (GD 9)	↓ TSPO staining (PFC) Similar TSPO staining in microglia, astrocytes and vascular cells [64]
**C57BL/6 mice** single (GD 9.5)	♂ **:** ↑ Microglia clustering, ↑ microglia processes, ↑ dark microglia, ↓ arborization area, ↓ phagocytosis (less cellular inclusions) (hip) ♀ **:** ↑ Microglia contacts with myelinated neurons, ↑ microglia processes, ↑ phagocytosis (more cellular inclusions) (hip) [126]
**C57BL/6 mice** single (GD 9.5)	♂**:**↑ CD11b ♀ **:** ↑ CD11b with CD68 puncta (hip) [127]
**Balb/c mice** single (GD 9)	♂ **:** ↓ CD11b and CD45 ♀ **:** ↓ CD11b (all brain) [129]

*ED* embryonic day, *PND* post natal day, *SCC* supraventricular corpus callosum, *CC* corpus callosum, *hip* hippocampus, *PFC* prefrontal cortex.

**Table 4:** Microglia sequelae: evidences from neurodevelopment until adulthood in others schizophrenia animal models.

		
Other schizophrenia animal models	Adolescence		Adulthood	
	PND 28	PND 35	PND 56	PND 60–100
**Wistar rats** LPS injection GD 17 until delivery	No changes in density (hip) ♂ and ♀ [112]	–	–	–
**Wistar rats** single poly I:C injection PND5-7	–	↑ Microglia activation ↑ Inflammation (PFC, striatum and hip) [116]	–	↑ Microglia activation ↑ Inflammation (PFC, striatum and hip) [116, 124]
**Gunn and Wistar rats**	–	–	Only ♂ ↑ Microglia activation Similar microglia density Microglia with enlarged areas of cytoplasm rich in organelles and some phagocytic pouches (hip) [121, 122]	–

*PND* post natal day, *PFC* prefrontal cortex, *hip* hippocampus.

### Prenatal period

In the prenatal period, microglia are actively proliferating and migrating to the appropriate regions of the brain [100]. After this period of brain colonization, microglia are engaged in the active regulation of the number of newborn neurons and/or synapses, according to their functional state [100]. This function of microglia is mainly supported by their phagocytic ability and by the production and secretion of molecular mediators, able to influence neuronal/synapse fate [43, 101, 102].

As previously mentioned, in the studies found in the literature, maternal immune activation model is the main experimental option to model schizophrenia when the main aim is to evaluate microglia. Although we found different protocols of poly I:C administration (different gestational days, GD, single shot or double injection), a factor that certainly interferes with brain development, the fact is that the reported microglia changes are consistent between studies.

When the administration of poly I:C is performed early in gestation (GD 9.5), the offspring (embryonic day, ED, 17) presents transcriptomic alterations in microglia, particularly in genes associated with cell motility structures (protrusions) and neuritogenesis [103]. The administration some days later (GD 12.5 or 14.5) accelerates the establishment of a transcriptional profile (assessed in ED 14) characteristic of later developmental phases [34]. Poly I:C administration at mild/mild-late gestation period (at GD 12 or 15), triggers an increase in microglia dynamics (total distance moved over time), without interfering with morphology (evaluated at ED 18) [104]. A single or double injection of poly I:C (at GD 11.5 and/or GD 15.5) did not alter microglia density or the levels of pro-inflammatory cytokines at ED 17.5 [105].

In summary, apart differences in the maternal immune stimulation plan, this prenatal stimulus produces congruent changes of microglia transcriptome, mainly associated with the early acquisition of a profile typical from a later stage (one study report alterations in motility that, however, do not impact on colonization or cell density).

### Postnatal period

Maternal immune activation during gestation, besides the alterations already observed *in utero*, is also associated with changes in microglia density after birth. However, the published works analyze different brain regions upon exposure to poly I:C at different gestational periods, protocol details that hinder concluding about increases or decreases in density. Microglia density per brain region may be expressed as the total number of cells or as the number of specific cellular phenotypes, namely amoeboid (in perinatal phases indicates greater immaturity) or already equipped with protrusions or cellular processes and ramifications.

The administration of poly I:C close to the delivery day (GD 20) is associated to an increase in the total density and in the density of amoeboid microglia in the hippocampus, immediately after birth, that is, at postnatal day (PND) 1 [106]. Earlier administration of poly I:C (mild-late administration, GD15) increased the number of amoeboid microglia in supraventricular corpus callosum (region where primitive microglia accumulate and then migrate tangentially across corpus callosum, acquiring a ramified phenotype), while associated with a decrease in corpus callosum, striatum, somatosensory cortex and hippocampus [107], suggesting an effect upon density dependent on the brain region under analysis. It is important to note that changes in microglia density, in this period of development, can result from changes in proliferation, death or migration; to disentangle which specific mechanism drives microglia changes may guide assertive therapeutic options.

In addition to changes in microglia density, alterations in the systems CX3CR1-CX3CL1 and CD200R-CD200 (evaluation of mRNA and protein levels of the ligands and respective receptors) were observed in the cortex and in the hippocampus of the offspring of mothers exposed to poly I:C (GD 15) or to a different immune stimulus such as lipopolysaccharide (LPS), at GD7 [108]. These two systems are important hubs of communication between microglia and neurons, and the described alterations may suggest an impairment in the crosstalk between different types of cells, that is fundamental to adequate circuit wiring and development.

### Adolescence

Adolescence, a period of intense plasticity and neuronal circuit rearrangements, is also a critical time window of susceptibility to brain disorders, including schizophrenia [109]. Moreover, schizophrenia diagnosis is often associated with adolescence, the driver for pre-clinical studies in this phase of life using animal models of the disease.

In the period of rodent weaning (PND 21) no changes were observed in the total number or in the number of phagocytic microglia [110]. However, a different study reports an increase in the number of amoeboid cells in the hippocampus of male descendants of rodent mothers subjected to poly I:C administration at GD 15 [111]. Again, the moment of poly I:C administration is different (GD 9 when no changes were observed) between studies.

A few days later (PND 28), LPS offspring (injections from GD 17 until delivery) still not present changes in microglia density in the hippocampus [112]. Nevertheless, 2 days later, poly I:C offspring (PND 30) present a higher number of microglial cells in the hippocampus and striatum, but not in the frontal cortex. This increase was accompanied by morphologic alterations (e.g. reduced number of processes and branches) only in the hippocampus [113].

More recently, it was observed that morphologic changes parallel changes of functional markers in the poly I:C model, and described enlarged microglial cell bodies and a retraction of processes (alterations often taken as indicators of a reactive state, typically associated to disease conditions) in the hippocampus, corpus callosum, striatum, and PFC, accompanied by an increase in the expression of inducible nitric oxide synthase (iNOS), a key enzyme involved in inflammatory processes [114].

In line with the present knowledge about microglia heterogeneity throughout the healthy brain, but also in psychiatric conditions [40–42, 89], differences found in microglia at adolescence are likely dependent on the brain region under study. Interestingly, at this age, sex-dependent changes in microglia immune profile were also observed in poly I:C offspring (only females present alterations in genes related to a pro-inflammatory state, namely an up-regulation of interleukin 4 receptor (CD124) and macrophage mannose receptor (CD206) and a down-regulation of Cluster of Differentiation 54 (CD54), C-C chemokine receptor type 2 (CCR2, CX3CR1) [115]. This observation is particularly meaningful and aligned with the work developed in our lab, which is focused in sex-specific morphologic remodeling processes in psychiatric diseases [40–42, 89, 91].

The results gathered so far, highly suggestive of a pro-inflammatory profile of microglia in the period of adolescence, are corroborated by studies with different designs. For instance, if the administration of poly I:C is performed after birth (e.g. at PND 5-7), a pattern of microglia reactivity by a pro-inflammatory response is still observed in the hippocampus, striatum and PFC, at PND 35 [116]. Concordantly, others showed that the increase of pro-inflammatory cytokines in the hippocampus and PFC of poly I:C offspring (early administration, GD 9) at PND 40 is paralleled by the increase of microglia numbers in these brain regions, thus suggesting that microglia are probably a major player in this immune response [117].

Despite the consistency of the presented studies, one study reported the absence of microglia changes (in terms of number, morphology and phagocytosis) in the hippocampus of poly I:C offspring at PND 40 [110]. Controversial evidences were found in the number of microglial cells in the hippocampus of poly I:C offspring at PND 40 (even though the coincident administration of poly I:C at GD 9), but the studies were conducted in different animal species, presenting temporal differences in the appearance of some phenotypic characteristics (in fact, rat development lags ∼1.5 days behind mice [99]). In addition, the markers used to stain microglia in these studies were also different (IBA-1 versus CD68) and stain different microglia populations (IBA-1 – total microglia; CD68 – phagocytic microglia).

Finally, to further support the contribution of microglia for the pathophysiology of schizophrenia in rodent models and, in particular, during adolescence (PND 21-42), the depletion of these cells and the subsequent repopulation ameliorates several phenotypic traits of the disease. Particularly, poly I:C offspring presented changes in microglia transcriptome early in development (E17 and PND 7) that were no longer observed after microglia depletion and repopulation during adolescence, therefore contributing for the recovery of microglia-neuron communication and behavioral changes in this animal model at adulthood [103]. Additionally, the administration of minocycline (antibiotic with anti-inflammatory effects, usually used as a microglia modulator) during adolescence (PND 21-35) was also able to normalize the hyper-ramification of microglia in DG, as well as behavior alterations observed in adult mice prenatally exposed to poly I:C [118].

In summary, the data gathered so far at adolescence point to a general increase in microglia density, accompanied by a shift to a pro-inflammatory phenotype, typically associated to pathological conditions. The contribution of these alterations to the presentation of schizophrenia in the animal model is strongly supported by the elimination/repopulation experiment and is clearly suggestive of the relevance of microglia at adolescence to the pathophysiology of schizophrenia.

### Adulthood

Similarly to what happens in other time windows of rodent life, at adulthood, the majority of the studies available report alterations in the density, as well as morphologic remodeling of microglia and a deviation to a pro-inflammatory state, commonly referred as activation state and often related with pathologic conditions or threats to homeostasis.

At adulthood (PND 56), higher levels of a TSPO radiotracer were detected in the PFC and hippocampus of rats prenatally exposed to poly I:C (early gestational period, GD 9) [119]. Conversely, others describe a decrease in TSPO staining in the PFC (but not in the hippocampus) in the poly I:C model [64]. As previously explained, caution must be taken when considering TSPO levels as an index of neuroinflammation mediated by microglia or even microglia activation, since other cells, including astrocytes and vascular endothelial cells, also express TSPO [64] and it was recently demonstrated that its expression does not correlate with the expression of microglia activation markers [65].

Other studies support a pro-inflammatory profile in the adult brain of schizophrenia models, gathering evidences for the increase in microglia density in different brain regions without [120] or with the presence of phagocytic structures compatible with the so-called activated state [121, 122]. Two studies performed later (PND 60–69) with rats prenatally [123] or postnatally [124] exposed to poly I:C further support the general idea of higher density and the presence of activated microglia. Curiously, one of these studies refers a reduction in the phagocytic ability of microglia [123] and other a pro-inflammatory environment in the PFC of rats subjected to prenatal poly I:C [117, 125], thus suggesting that microglia are predominantly in a pro-inflammatory state.

Interestingly, at PND 80–90, sex-specific changes in the density (more microglia clusters in the hippocampus of males), ramification level (lower ramification in the hippocampus of males), phagocytic activity (lower phagocytic activity in males) and interaction with synapses (more contact points with neurons in the case of females) have been described in the poly I:C model [126]. Recently, the same authors demonstrated that these phenotypic alterations are associated to sex-specific changes in excitatory and inhibitory synapse density. Poly I:C males present more synapses and inhibitory inputs, probably due to a deficient synaptic elimination, whereas females present a reduction in the number and activity of excitatory synapses [127]. These studies are highly relevant in the sense they describe a functional disturbance of the neuronal network, possibly correlated with poly I:C-induced microglia phenotypic alterations. These observations are in line with already described failures in the communication between microglia and neurons, which depends on the ability of microglia to sense their local environment. In fact, Mattei and co-workers described changes in a cluster of genes involved in microglia sensing abilities (sensome) [123]. Further suggesting a deficiency in microglia-neuron communication in schizophrenia models, a study demonstrated a disruption in the CX3CL1 (fractalkine released by neurons)-CX3CR1 (fractalkine receptor expressed by microglia) axis in poly I:C animals [128]. Finally, Manitz and co-workers proposed an impairment of the proper surveillance of brain parenchyma by microglia, based on the detection of alterations in microglia markers, namely a decrease of CD11b (integrin of the complement receptor 3) levels in poly I:C offspring at PND 100 (at both sexes) and a decrease of CD45 (negative regulator of microglia activation), exclusive to males. As described in other models of psychiatric disorders, these observations suggest that microglia sequelae are dependent, not only on the brain region under analysis, but also on sex [129], a question that deserves further investigation, considering differences in the incidence and clinical presentation found in clinics.

In summary, at adulthood, the majority of studies point to increased microglia density and activation state associated with a pro-inflammatory environment, a pattern of alterations apparently starting during adolescence. Some evidences also demonstrate a functional compromise of microglia, associated with their phagocytic ability, an alteration potentially implicated in the incorrect selection of synapses for pruning and behavior abnormalities. Future studies should clarify this issue.

### Main conclusions and implications for schizophrenia diagnosis and therapeutics

Innate immunity and, in particular, its cellular elements (typified by microglia in the central nervous system), have been implicated in several psychiatric diseases with genesis during brain development. In early development, the fine regulation of synapse formation and selection for elimination is assisted by microglia-mediated immune functions, subsequent to the proper detection of neuronal/synaptic molecular signs. Thus, any brain or peripheral alteration detected by microglia may elicit a response with potential to interfere with the normal course of development, with impact in the number, degree of maturation and function of synapses/neurons and, ultimately, in behavior and health.

We find scattered studies exploring schizophrenia-associated changes in microglia cells, both in animal models of the disease (mainly obtained by gestational immune activation) and in patients. However, we were not able to find a review organizing and integrating pre-clinical and clinical information, to clearly characterize microglia changes associated to schizophrenia. The main aim of this work is to fill this gap, that we consider mandatory to rethink therapeutic targets and strategies for schizophrenia.

As main conclusion, schizophrenia-associated microglia sequelae are consistently observed in patients and animal models of disease, even considering the lack of consistency in the design of clinical and pre-clinical studies, that generates apparently conflicting data.

Clinical findings, mainly obtained by neuroimaging (PET analysis), are biased by a serious of confounding factors that must be considered in future studies. These factors include, mainly but not exclusively, individual and inter-individual variability associated to: sex and age of onset, predominance of positive or negative symptoms, type and duration of pharmacological treatment of schizophrenia and/or comorbidities. It is important to have in mind that any immune-inflammatory concomitant condition may affect microglia and this is a critical aspect, often undervalued in the literature. In addition, there are important technical limitations in assessing microglia or microglia correlates in live patients. For instance, a variety of different tracers (with variable affinities for endogenous ligands and erroneously taken as microglia markers) are used in PET, and a clear definition of the outcomes to be analyzed must be rethought and implemented. The clear meaning of normal, increased or decreased PET tracing must be reinterpreted in a consensus evaluation of its pathophysiological implications and correlation with the clinical presentation of schizophrenia (including the correlation suggested by some studies with the predominance of positive or negative symptoms).

The post-mortem study of microglia from schizophrenia patients globally demonstrates: (1) transcriptomic changes; (2) density variations – increase density in frontal and temporal cortices and a lateralization effect, that deserves further investigation; (3) the presence of amoeboid microglia in frontal and temporal cortices and in the hippocampus (Table 2), apparently more evident in the case of positive symptoms predominance (this finding reinforces the importance of establishing rigorous criteria in patient’s stratification). As claimed for in vivo studies, microglia markers used in post-mortem studies present some limitations, namely in selectivity (an overestimation of microglia alterations may result from the contribution of other cells also stained by the same markers, including perivascular macrophages and/or infiltrating peripheral macrophages). Finally, one cannot ignore the contribution of the cause of death to microglia changes, as well as the influence of time of post-mortem, aspects to be considered in the design of future studies.

Animal models of schizophrenia, namely based on the immune stimulation during gestation, are very important sources of information, otherwise impossible to get from clinical studies. It is important to note that the main data from animal studies presented and discussed in this article were obtained modeling schizophrenia by maternal immune stimulation. As previously mentioned, this animal model presents neurochemical changes (e.g. dopamine dysfunction from early development – for review see ref. [130]) and behavioral traits characteristic of schizophrenia (as well as of other developmental brain diseases, including autism, with similar symptoms and risk factors; for detail see refs. [131, 132]). So, it is of importance to further study microglia in alternative models to establish a universal mechanism of disease and disease manifestations. From different pre-clinical studies, it is unquestionable that microglia undergo alterations associated with schizophrenia modeling. Overall, microglia alterations will vary according to the period of life under analysis and can include changes in: transcriptomic profile, motility, migration/colonization, activation state (including morphologic alterations and cytokine release) and function (phagocytosis, crosstalk with neurons; Table 3 and Fig. 3). Prenatally, most changes involve the transcriptome, with microglia acquiring an adult-like phenotype earlier than in basal conditions. The main alterations described in microglia at postnatal period rely on density (increase) and migration (delay). At adolescence, the increased density is still present and is accompanied by a pro-inflammatory environment (characteristics typically associated with the so-called activated microglia). Notably, adolescence seems to be a critical period for intervention, since microglia elimination/depletion or modulation (by the control of the pro-inflammatory environment through minocycline treatment) rescue microglia transcriptomic and morphologic alterations, as well as microglia-neuron communication, in parallel with the recovery of behavior traits typically present in schizophrenia models. At adulthood, microglia still present an activation state and some functional issues emerge, namely the impairment of phagocytic ability and of microglia-neuron communication.

According to the information organized in the present work, it is clear that microglia is involved in the pathophysiology of schizophrenia. If, as suggested by pre-clinical research, the normalization of disease-associated changes of microglia, is also able to ameliorate human symptoms of the disease, it is important to design future studies including immune modulation strategies able to limit microglia numbers, inflammatory phenotype and/or phagocytosis. In this regard, considering the lack of efficacy of anti-inflammatory compounds in symptoms remission (corticosteroids or non-steroid anti-inflammatory drugs, already tested in clinical trials (https://clinicaltrials.gov/), it would be desirable to improve the selectivity towards microglia. In parallel, it is also mandatory to clarify if and how the immune modulatory effects of antipsychotics impact on microglia and if these eventual effects contribute to drug efficacy. The limitations associated to neuroimaging data highlights the need of more adequate tools to evaluate schizophrenia-associated microglia sequelae, that may include data from human iPSC-derived glia (as reviewed in refs. [133, 134]).

**Fig. 4:**
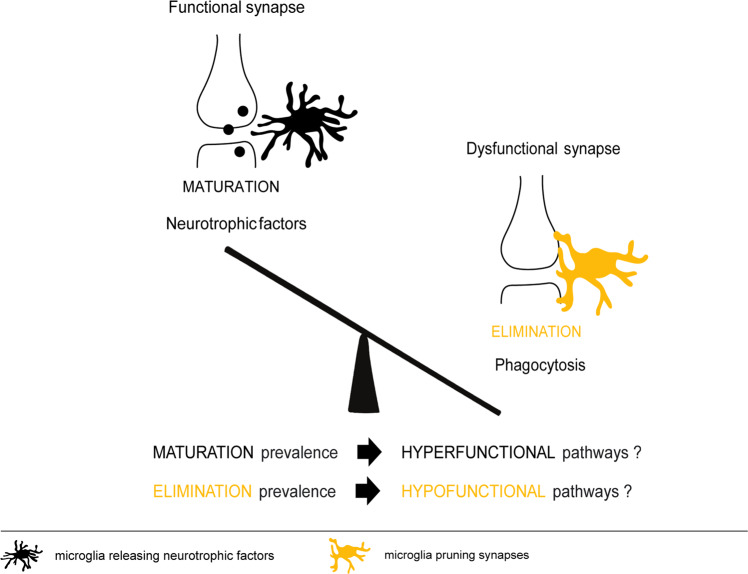
Putative mechanisms by which microglia may contribute for the establishment of dual dopaminergic pathways. Schematic representation of novel putative mechanisms by which microglia may oppositely influence dual dopaminergic pathways: imbalance between the ability to eliminate and strengthen synapses.

Far behind symptoms manifestation and diagnosis, we consider it is also important to better understand schizophrenia genesis during development. If, as suggested in the literature, an excessive synaptic pruning may be the kickoff for neurobiological deviations from normality, we think alternative mechanisms of disease need to be explored in order to explain the described hypodopaminergic function, for instance, deficits in synaptic pruning in these particular pathways. Synaptic pruning is a cellular response to local cues in the brain, thus, synaptic pruning may be differently affected in different brain areas. We hypothesize that the main pathways implicated in the disease, which are dually hypo- or hyperfunctional, may result from the excessive (as already described) or from the compromised (a hypothesis not tested so far) synaptic pruning (Fig. 4).

In conclusion, very early in development, a brain-region determined microglia defect could trigger different mechanisms of disease (kickoff). On the other hand, subsequent microglia changes (sequelae) likely persist during disease progression and it is apparently sufficient to target these sequelae to ameliorate psychotic symptoms. The second approach is relatively easy to test and implement in patients, but preventing kickoff mechanisms would require advanced knowledge about microglia mechanisms of pruning at the molecular level and *in utero* immune or genetic modulation of those mechanisms. Recent discoveries on the involvement of specific components of the complement system in microglia-mediated synaptic pruning clearly helps defining new therapeutic targets.

## Data Availability

Data sharing is not applicable to this article.
